# Patients’ Opinions on Antibiotics in the Treatment of Dental Infections: A Cross-Sectional Survey

**DOI:** 10.3390/jcm13072099

**Published:** 2024-04-03

**Authors:** Laura Domínguez-Domínguez, Pablo Castelo Baz, Alberto Cabrera-Fernandez, Daniel Cabanillas-Balsera, Manuel Pabon-Carrasco, Juan Jose Segura-Egea, Jenifer Martin-Gonzalez

**Affiliations:** 1 Department of Stomatology, Endodontic Section, School of Dentistry, University of Sevilla, 41009 Sevilla, Spain; lauradguez96@gmail.com (L.D.-D.); albertocfdez@gmail.com (A.C.-F.); danielcaba@gmail.com (D.C.-B.); 2 Oral Sciences Research Group, Endodontics and Restorative Dentistry Unit, School of Medicine and Dentistry, Health Research Institute of Santiago de Compostela (IDIS), University of Santiago de Compostela, 15706 Santiago de Compostela, Spain; pablocastelobaz@hotmail.com; 3 Department of Nursing, Faculty of Nursing, Physiotherapy and Podiatry, University of Sevilla, 41004 Sevilla, Spain

**Keywords:** antibiotic, antibiotic resistance, patients, automedication

## Abstract

**Background**: The aim of this study was to evaluate patients’ knowledge and perceptions of the use of systemic antibiotics in the treatment of endodontic infections and to determine the possible contribution of patients to the development of bacterial resistance. **Methods**: A total of 550 patients were asked to respond to a survey on the perception of systemic antibiotic use in the treatment of endodontic infections and antibiotic resistance during January 2022 and March 2023. A bivariate and multivariate analysis was performed to determine possible correlates in the population regarding antibiotic use in the endodontic world. **Results**: A total of 514 patients were included in the study, 65.9% of whom were women. While 34.6% of the population studied thought that it was always necessary to take antibiotics prior to endodontics, 49.4% considered that they were necessary after endodontics, regardless of the clinical symptoms. The prevalence of self-medication was 17.3%, and women self-medicate more than men, with significant differences (*p* < 0.05), although they have a greater knowledge of antibiotic resistance than men (*p* < 0.05). Forty-four percent of the population expected to take antibiotics when faced with dental pain, mainly women (*p* < 0.05). **Conclusions**: The general population is contributing to the serious problem of bacterial resistance. It is necessary to promote educational strategies focused on the correct use of antibiotics in the community. The worst results were found mainly in the population with a low level of education. The level of education was the variable that most influenced the knowledge and attitudes of the population, followed by the sex of the participants.

## 1. Introduction

One of the most serious threats facing Spanish and global public health is the emergence of infections caused by bacterial strains resistant to antibiotic treatment. Today, there are bacterial species resistant to the full range of antibiotics currently available, which represents a potential medical disaster. The World Health Organization (WHO) has estimated that without coordinated proactive actions among all countries by 2050 there will be more deaths from antimicrobial resistance than from cancer [1].

The use of antibiotics in dental procedures represents 10% of the total prescriptions (a not insignificant percentage: one billion daily doses of antibiotics received by the population) [2]. The main factor responsible for this increase in resistance is inadequate prescribing by the health professional [3]. However, the factors influencing inappropriate use do not only involve healthcare personnel but also social policies such as a lack of control in the sale of antibiotics, a lack of knowledge and attitude of patients about the use of antibiotics and self-medication. In daily clinical practice, dental decisions are often motivated by the patient’s attitude [4,5,6,7]. Sometimes, pressure exerted by the patient or companion can lead the dentist to inappropriately prescribe antibiotics to please the patient. Therefore, it is very important that the general population is well aware of the role of antibiotics in the treatment of endodontic infections, as well as the implications that their inappropriate prescription has on the development of bacterial resistance. In addition, there are prescriptions “just in case” or out of fear of complicating the procedure [7].

For this reason, the European Commission undertook a series of surveys among the general population to monitor their awareness and levels of use [8,9]. There is evidence that antibiotic prescription in endodontics is inadequate [10]; however, there is no evidence of the population’s knowledge in this area. Some studies point to educational level as a variable to be taken into account in the fight against antibiotic resistance [11,12].

Therefore, the main objective of this study was to evaluate patients’ perceptions regarding the need to use antibiotics in endodontics to promote educational strategies focused on the correct use of antibiotics in the community.

The following hypotheses are proposed by the researchers as research hypotheses:

1. The population does not have sufficient knowledge for the adequate use of antibiotics and has a proactive attitude toward their indiscriminate use.
2. The patient profile is expected to be a male over 40 years of age with a low educational level.

## 2. Materials and Methods

### 2.1. Study Populations

The study was approved by the ethics committee of the University of Seville. (Research Ethics Committee Endodontic Postgraduate Course—University of Seville, number CEME-2/22). In this cross-sectional study, 550 patients from the Faculty of Dentistry of the University of Seville were included. They were asked to respond to a survey conducted on Google Forms regarding the perception of systemic antibiotic use in the treatment of endodontic infections and antibiotic resistance. Recruitment took place at the time of patient visits. The objectives of the study were explained to them. Those interested signed an informed consent form and were provided with the link to the questionnaire so that they could respond. The link was provided by e-mail. The survey was carried out between the months of January 2022 and March 2023. The inclusion criteria were patient adults with no possession of a medical or dental degree.

Respondents were classified according to their educational level (low, medium or high). The low level included persons with no education and persons in possession of compulsory primary education (school-leaving certificate); medium educational level persons included those with a compulsory secondary education and general baccalaureate. Respondents with a high educational level were considered people in possession of a certificate of professionalism, higher grade training cycle, university degree, university postgraduate degree and doctorate.

### 2.2. Questionnaire

The survey questions were adapted from those asked in a previous survey conducted in Barcelona on patient perception of antibiotic use after tooth extraction [7]. In addition, questions on antibiotic resistance were added. The questionnaire (Supplementary S1) was reviewed by dental researchers and professors of the Postgraduate Course in Endodontics at the University of Seville for the appropriateness and clarity of questions. Patients who participated in the survey did so anonymously, voluntarily and without compensation. The survey was satisfactorily completed by 514 of the 550 patients, who were subsequently included in the study.

The sociodemographic variables of gender, age of participants and educational level were collected. In addition, participants were asked if they had ever received a root canal (yes/no). The remaining questions in the questionnaire cover issues of attitudes and knowledge about the use of antibiotics. The appropriate answer is marked in bold.

Perception of the need to take antibiotics before and after endodontics treatment.

1. Before endodontics, do you think it is necessary to take antibiotics? (yes/**no**)
2. After a root canal, do you think it is necessary to take antibiotics? (yes/**no**)
  Participants’ perception and knowledge of dental infection.
3. If the dentist does not prescribe antibiotics, I would ask him/her why he/she does not prescribe antibiotics. (yes/**no**)
4. If the practitioner does not prescribe antibiotics, would you seek out another doctor to ask why your doctor does not prescribe antibiotics? (yes/**no**)
5. If a dentist tells you that you have a dental infection, would you expect him/her to prescribe antibiotics? (yes/**no**)
6. If you have dental pain, do you expect the dentist to prescribe antibiotics? (yes/**no**)
7. Have you ever self-medicated with antibiotics for dental pain? (yes/no)
  Benefits of antibiotic treatment.
8. What do you think are the benefits of antibiotics? You can choose more than one option. (Reduces pain, Reduces inflammation, **Reduces infection**, Improves oral health, No benefit, I do not know)
  Adverse effects of antibiotic treatment.
9. What adverse effects do you think the use of antibiotics can cause? You can choose more than one option. (**Nausea/vomiting, Diarrhea**, Fever, Fungal infection, **Allergic reaction**, None of the above, I do not know)
  Duration of antibiotic treatment.
10. When you take antibiotics, for how long do you take them? (1 day, 2 days, **3 days**, 1 week, 2 weeks, Other)
  Knowledge of respondents regarding antibiotic resistance.
11. Do you know about “antibiotic resistance” (a process caused by the overuse of antibiotics)? (**yes**/no)

Therefore, a participant with an adequate level of knowledge or a responsible attitude toward antibiotic use would be a participant who answers at least 60% of the questions correctly (8/13 questions).

The Cronbach’s alpha coefficient obtained was α = 0.770, indicating good internal consistency [13].

### 2.3. Data Collection and Statistical Analysis

The sample size was calculated to achieve a power of 0.95, with an alpha error of 0.05 and an effect size of 0.2 (test method: Chi-square test, G*Power 3.0.10, Franz Faul, University of Kiel, Kiel, Germany). A total of 400 participants were necessary. The sample size was increased by 30% to account for potential losses.

A first exploratory descriptive analysis was performed, including qualitative and quantitative variables. The distribution of variables was assessed using a Kolmogorov–Smirnov test. For hypothesis testing, a Chi-square test or Fischer exact test was used. For the quantitative variable, a Student’s *t*-test and ANOVA were used, as well as their nonparametric counterparts in case no requirements were met. All data were expressed as frequencies and their corresponding percentage. A multivariate regression was performed where the independent variable was the score of the participants. The dependent variables were considered to be the sex of the subjects, educational level and age.

All analyses were performed using SPSS ® VERSION 25.0, considering significant differences when *p* < 0.05.

## 3. Results

### 3.1. Participation and Description of Respondents

The demographic data of the 514 patients are described in Table 1. Female respondents (*n* = 339) accounted for 65.95% of patients and male respondents (*n* = 175) accounted for 34.1%. The majority of respondents were aged ≥ 40 years (*n* = 281, 54.7%). The educational level of the respondents varied widely, with a university degree being most prevalent (*n* = 150, 29.2%), followed by higher education (*n* = 111, 21.6%). Respondents with a high level of education represented 39.1% of the population (*n* = 201). Of the total number of participants, only 314 people (61.1%) had ever had root canal treatment (RCT).

### 3.2. Perception of the Need to Take Antibiotics before and after Endodontics Treatment

**Figure 1:**
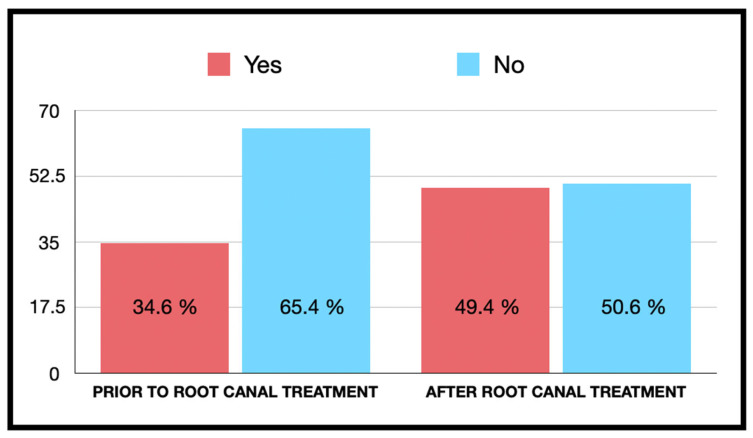
Belief about the need to take antibiotics before and after endodontic treatment.

Figure 1 represents the patients’ perceptions of the need to take antibiotics before or after RCT. On the one hand, there were 178 respondents (34.6%) who thought it was necessary to take antibiotics before RCT. Similarly, this situation increased to 254 respondents (49.4%) who considered it necessary after RCT, regardless of symptoms. Among the respondents who considered it necessary to take antibiotics before RCT, the majority were women (*n* = 112; 33%); however, there were no significant differences with respect to sex (OR = 1.23; 95% CI = 0.84–1.80; *p* = 0.291) or the age of the participant (*p* = 0.969). On the other hand, there were significant differences with respect to educational level (*p* = 0.04); specifically, correct and better responses were observed in participants with a medium and high educational level.

Similarly, among those who considered it necessary to take antibiotics after RCT, no significant differences were observed with respect to sex (*p* = 0.07) and age (*p* = 0.34). On the other hand, differences were observed with respect to educational level, with better results in the medium and high educational levels (*p* = 0.01). With respect to the previous experience of the participants, knowledge in the use of antibiotic therapy increased after the treatment (*p* = 0.001).

### 3.3. Participants’ Perceptions and Knowledge of Dental Infection

**Figure 2:**
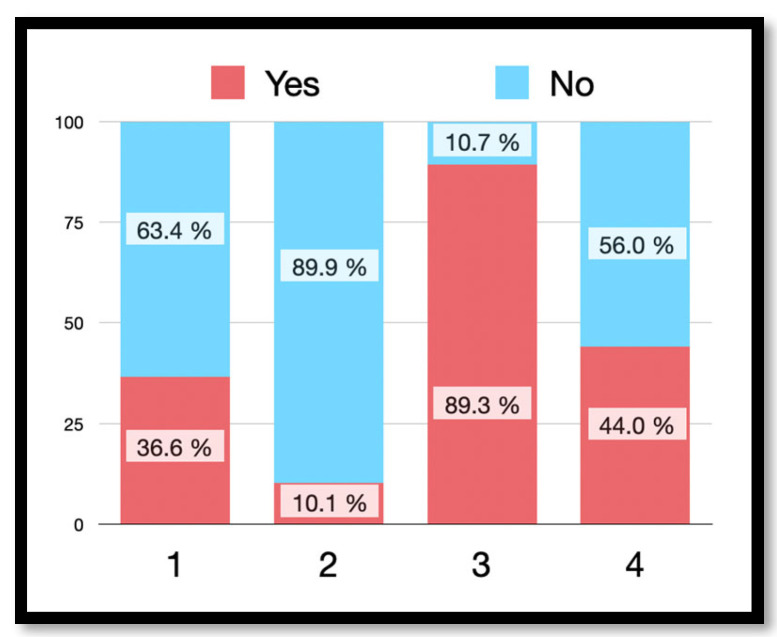
Perception and knowledge about dental infection. (1) If the practitioner does not prescribe antibiotics, would you ask why he/she does not prescribe them? (2) If he/she does not prescribe antibiotics, would you look for another doctor? (3) If your doctor tells you that you have a dental infection, would you expect him/her to prescribe antibiotics? (4) If you have dental pain, would you expect him/her to prescribe antibiotics?

Figure 2 shows the answers of the participants to different questions on dental infection and antibiotic therapy.

Given the clinical situation of having to receive a RCT, if the dentist does not prescribe antibiotics, 36.6% of respondents would ask why he does not do so, and 10.1% of the patients would even turn to another dentist to ask why antibiotics had not been prescribed (Figure 2). The mistrust of not prescribing antibiotics is related to a patient profile with a low educational level (*p* = 0.040) and under 30 years of age (*p* = 0.047), with no differences by gender (*p* = 0.240).

**Figure 3:**
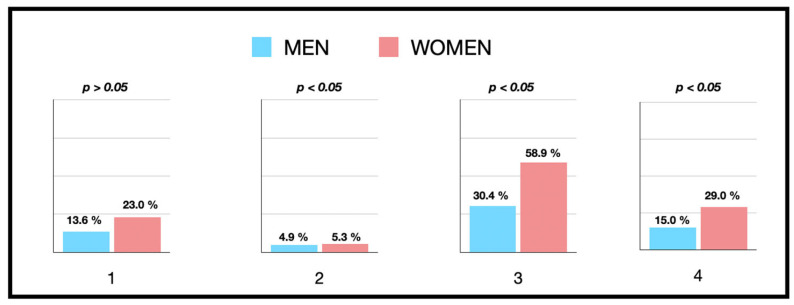
Perception and knowledge about dental infection according to gender with respect to the answer: Yes. (1) If the practitioner does not prescribe antibiotics, would you ask why he/she does not prescribe them? (2) If he/she does not prescribe antibiotics, would you look for another doctor? (3) If your doctor tells you that you have a dental infection, would you expect him/her to prescribe antibiotics? (4) If you have dental pain, would you expect him/her to prescribe antibiotics?

If we disaggregate the response by sex, men were more likely to seek a second opinion than women (women 8% versus men 14%; *p* = 0.024) (Figure 3). In addition, a higher second-opinion seeking was observed in patients under 30 years of age (*p* = 0.041).

Amongst respondents, 89.3% expected to receive antibiotic therapy to treat any dental infection (Figure 2), with no significant difference by sex or educational level. However, again the response rate was lower in patients under 30 years of age (*p* < 0.019) (Figure 3). Similarly, 44.0% thought that dental pain would be reduced with antibiotic therapy (Figure 2), with no difference being found by sex (Figure 3) or age. The low educational level considered a greater need to take antibiotics for pain than the medium or high educational level (*p* = 0.001, 63.8% low educational level versus 45.6% medium educational level or 31.8% high educational level).

Regarding self-medication, 89 respondents (17.4%) had self-medicated with antibiotics without a medical prescription on at least one occasion. Men (22.3%) had self-medicated more for dental pain compared to women (14.7%), with statistically significant differences (OR = 1.66; 95% CI = 1.04–2.64; *p* = 0.032). A significant relationship was observed between self-medication and seeking a second opinion in both men and women, especially those over 40 years old (*p* = 0.001). Individuals with a medium or low educational level self-medicated more than those with a high educational level, with statistically significant differences (OR = 0.99; 95% CI = 0.99–2.52; *p* = 0.018). On the other hand, age was not a determining factor in self-medication (*p* = 0.158).

### 3.4. Benefits of Antibiotic Treatment

**Figure 4:**
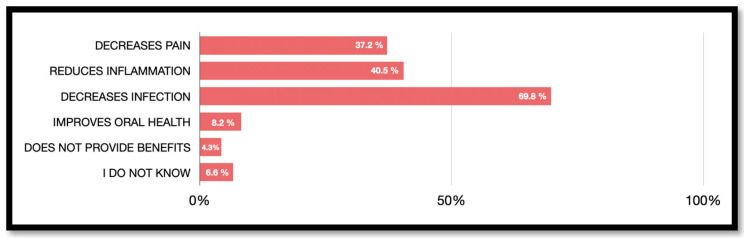
Beliefs about the potential benefits of antibiotic therapy.

Participants could select more than one option regarding the benefits of antibiotics, the correct answer being a reduction in infection. The respondents’ beliefs about the potential benefits of antibiotic therapy are shown in Figure 4. A reduction in infection (*n* = 369, 69.8%) was the most selected benefit, followed by a reduction in inflammation (*n* = 208, 40.5%) and a reduction in pain (*n* = 191, 37.2%). There were no significant differences regarding sex, age and level of education (*p* > 0.05). With respect to age, it is observed that those over 40 consider that the use of antibiotics reduces the risk of infection (≤30 years 21.7% versus 30–40 years 17.0% versus ≥40 years 61.3%; *p* = 0.001). This constatement is repeated repeatedly in other questions such as pain reduction (*p* = 0.001), inflammation reduction (*p* = 0.001) and improved oral health (*p* = 0.050). With respect to the previous experience of the participants, the knowledge of the use of antibiotic therapy to reduce post-treatment infections increased (*p* = 0.002).

### 3.5. Adverse Effects of Antibiotic Treatment

**Figure 5:**
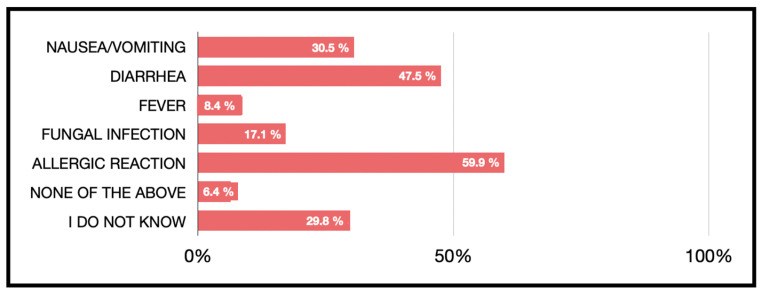
Beliefs about the adverse effects of antibiotic therapy.

The answers to the question about the adverse effects of antibiotics are shown in Figure 5. Diarrhea (47.5%) was the adverse effect most cited by respondents, followed by allergic reaction (40.1%) and nausea/vomiting (30.5%).

Regarding sex, women were more likely to consider diarrhea (*p* = 0.001, 54.9% women versus 33.1% men) and fungal infection (*p* = 0.001, 20.9% women versus 9.7% men) than men. Also, it was observed that men had a greater ignorance than women (“I do not know” response *p* = 0.001, 24.8% women versus 39.4% men).

Regarding age, those younger than 30 years old considered antibiotics to have a higher percentage of adverse effects such as fever (*p* = 0.001), allergic reactions (*p* = 0.001) and nausea/vomiting (*p* = 0.001) with respect to those older than 40 years old.

Finally, with respect to educational level, it was observed that participants with a higher level of education had a higher frequency of response for side effects in allergic reactions, fungal infections, diarrhea and nausea/vomiting. (*p* < 0.01).

### 3.6. Duration of Antibiotic Treatment

**Figure 6:**
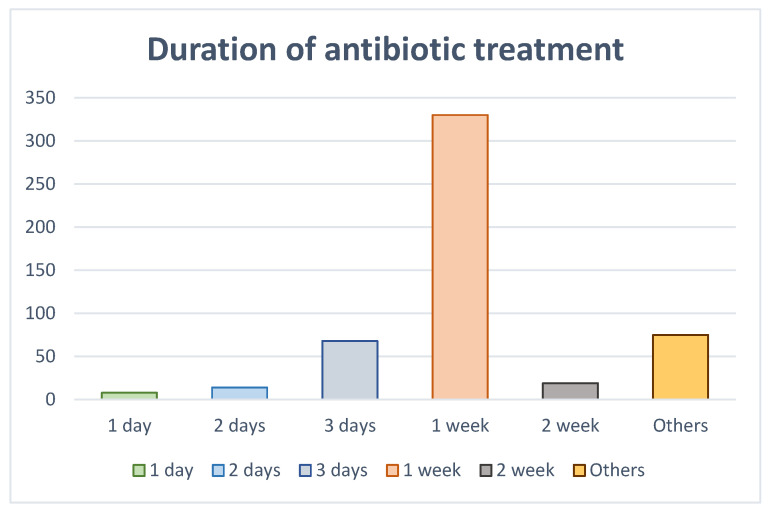
Respondents´ beliefs about the duration of antibiotic treatment.

When asked about the duration they believed antibiotic treatment should last, the majority of respondents (*n* = 330, 64.1%) considered that it should be one week, and 13.2% (*n* = 68) considered that it should be 3 days (Figure 6).

A difference with respect to sex was observed, with women believing more strongly in extending the guideline by one week (women 68.1% vs. men 56.6%; *p* = 0.06). In terms of age, the highest percentage of those over 40 years of age believed in prolonging the antibiotic regimen at least one week (≤30 years 23% vs. 30–40 years 16.1% vs. ≥40 years 60.9%; *p* = 0.001). If we segmented the sample by sex, we observed that those under 30 years of age had better information in both sexes regarding the duration of treatment (*p* = 0.001).

### 3.7. Knowledge of Respondents Regarding Antibiotic Resistance

Finally, respondents were asked about antibiotic resistance. Approximately half of those surveyed (*n* = 274, 53.3%) responded that they were aware of the problem of antibiotic resistance caused by antibiotic overuse. There were no significant differences in the responses to this question in terms of sex and age (*p* > 0.05). However, the level of education did influence the knowledge of antibiotic resistance. The higher the level of education, the better the perception of the concept of antibiotic resistance (*p* = 0.0001). If we segmented the sample by sex, we observed that a high educational level was significant in both men and women (*p* = 0.001).

### 3.8. Multivariate Analysis of Attitudes and Knowledge with Respect to Sex, Age, Educational Level of the Participants and Previous Endodontic Experience

A multivariate analysis was performed with the independent variable being the final scores of the participants (maximum score of 13 points). Specifically, a logistic regression was performed where the independent variable was the level of knowledge (insufficient scores lower than 8 points or sufficient scores higher than 8 points). Higher scores were observed for people with a high level of education and whose gender is female. Generational age was not a significant variable in the final score (Table 2).

In the model, it can be observed that a high educational level (OR 2.24 IC 95 1.366–3.684) together with the female gender (OR 1.99 IC95 1.366–1.506) significantly influences the knowledge of the participants. The rest of the variables are removed from the model because they are not significant (age and previous experience in endodontics).

## 4. Discussion

The results of the present study, analyzing the responses to the survey of 514 people, demonstrate the lack of knowledge of the patients about the use of antibiotics in the treatment of endodontic infections. It was observed how the level of education has a significant implication on attitudes and knowledge regarding the use of antibiotics in dentistry. These results are consistent with previous studies [11,12]. Furthermore, it is possible to define a profile based on the different areas evaluated. On one hand, we find better general knowledge in all facets in individuals with a high level of education, regardless of the participant’s gender. Regarding gender, men are much more skeptical than women, with women being better at detecting side effects than men, especially if they are under 30 years old. Additionally, it was observed that men self-medicate more frequently, a situation that is highly correlated with their level of distrust (they would seek a second opinion and inquire why antibiotics are not prescribed). Generational age is the variable that least influences, along with previous endodontic experience.

This study reflects the current state of knowledge and patient perception of antibiotic resistance and the use and need for systemic antibiotics in the treatment of pulpo-periapical infections. Previous studies [2,10,14,15,16,17] have evaluated dentists’ knowledge of antibiotic-prescribing habits. However, although it is known that dentists’ prescribing habits are sometimes influenced by patient pressure, until now, no study had analyzed patients’ knowledge and beliefs about antibiotic treatment in endodontics. Only a previous study had analyzed patients’ beliefs about antibiotic treatment in cases of tooth extraction [7].

The sample of this study was representative of the general population, and the overall response rate was high (93.4%), larger than other studies [7].

Several studies have investigated self-medication with antibiotics [4,7,18,19,20,21,22], analyzing the impact of predisposing factors, such as knowledge and perception of antibiotic use, and facilitating factors (wealth of the country and health system factors). A survey carried out in 19 European countries showed that Spain together with Lithuania and Romania were countries with a very high rate of self-medication. Moreover, these three countries together with Italy were the countries where many antibiotics were accumulated at home [4,22]. Antibiotic dispensing by tablet size can generate leftovers that contribute substantially to self-medication [4]. In addition, medications may be left overdue to patient noncompliance, as the patient may not take the prescribed amount of medication and subsequently self-medicate. It has been shown that 36% of people who self-medicate do so using leftovers [4,22]. In Spain, antibiotics are not dispensed by exact number but by tablet size. In Spain, self-medication is a daily occurrence; a 2008 study showed that 18.1% of all Spaniards self-medicate [5]. According to the results of the present study, 17.3% of respondents have self-medicated on occasion with antibiotics without a prescription. These results are similar to those previously described in Spain in 2008, in Portugal [23] and in southern Europe [4]. Regarding the relationship between self-medication and gender, men self-medicate more when faced with dental pain than women (OR = 1.7; *p* = 0.032). The findings of the current study do not align with those of previous studies conducted in Spain and other countries [5,20,21], which found that women had a higher exposure to medication consumption than men. Conversely, they align with the results of the study conducted in Portugal [23], which showed a higher prevalence rate of self-medication in men. This difference could be explained by the different compositions of the samples in both studies, with different proportions of men and women. However, it is worth noting that our study is homogeneous with respect to age, previous experience in endodontics and educational level. Regarding educational level, the percentage of self-medication is higher among individuals with a lower level of education, with statistically significant differences found (*p* = 0.018). Grigoryan et al. (2006) [4] found similar results. It would be expected that patients’ attitudes reflect their level of health literacy, which in turn may be related to education [24].

Regarding age, the results of the present study have not shown it to be a determining variable. These data do not align with the findings of previous studies where the highest prevalence of self-medication was found among younger age groups [5,22,23].

However, the present results, showing that older patients with a higher educational level had a more conservative perception of the need for antibiotic treatment, are in good agreement with those of Pérez-Amate et al. [7].

On the other hand, the high percentage of patients who if not given antibiotics would inquire why or would seek a second opinion if the first healthcare provider did not prescribe antibiotics not only highlights the population’s lack of knowledge but also demonstrates the level of distrust toward healthcare professionals and the social pressure under which professionals are placed. This was even more evident in the profile of young patients with lower levels of education, regardless of gender.

More than half of the participants think that antibiotics reduce pain, and these results could be explained by the lack of updating of the health professional [14,15,17,25,26] and the lack of time that the health professional sometimes dedicates to explaining to patients the need or not for antibiotic treatment. Pain is not always synonymous with infection, which is why it is necessary to explain to patients the post-intervention process, making clear the warning signs as well as the natural process after an intervention. It is therefore essential that the dentist spends more time explaining to patients the reasons why they do not need antibiotics in each clinical situation [7].

To determine the general population’s knowledge of antibiotic use, different options of the possible benefits of antibiotics were included in the survey, including a reduction in pain, reduction in inflammation, reduction in infection, improvement in oral health, no benefit and I do not know. A reduction in infection (69.8%) was the most selected benefit, in agreement with the results obtained in the previous study performed in Barcelona [7].

According to clinical guidelines [27], the duration of antibiotic treatment should be based on the improvement in the patient’s symptoms, such that antibiotic therapy will last until symptoms have resolved. However, almost two-thirds of the respondents considered that they were necessary for 1 week. Significant differences were evident in relation to age and gender. Women were even more likely to believe that antibiotics last for one week. This was more plausible in women over 40 years of age. On the other hand, only 13.2% considered the duration of antibiotics to be 3 days. Surveys previously conducted with patients did not analyze their perception regarding the duration of antibiotic treatment. The present results again highlight that the misconception that bacterial infections require “a full course” of antibiotic therapy still persists, but no evidence that a one-week period is necessary to treat endodontic infections has been found [27].

Patients’ erroneous knowledge regarding antibiotic treatment contributes to the misuse of antibiotics and, consequently, to the problem of the development of bacterial resistance. Even though there are currently many programs in place, both globally and internationally, to curb the increase in bacterial resistance, the results of the present study show that only approximately half of the patients (53%) are aware of the problem of bacterial resistance to antibiotics. In the study carried out in 2021 in Barcelona [7], the patients’ knowledge of antibiotic resistance was higher (69%). The present results showed that there were not significant differences in relation to age and sex, but there were significant differences in relation to the educational level of the respondents. This highlights the need for campaigns to improve knowledge regardless of the educational background of the patient.

This study is not without its limitations. On the one hand, cross-sectional descriptive studies cannot perform causality; they only describe a specific situation, thus undermining external validity. This situation has been overcome by increasing the sample size. On the other hand, despite the high internal consistency of the scale, it is necessary to validate scales that allow us to collect the behavior of users in the use of antibiotics in the dental field. In this way, we could have cut-off points that allow us to determine the degree of knowledge and thus be able to carry out specific actions in vulnerable populations or those with little information.

## 5. Conclusions

The results of the present study show the lack of knowledge of the general population on the use of systemic antibiotics in the treatment of endodontic infections. It is necessary and essential to develop new educational strategies to improve the knowledge and perception of the Spanish population on the correct use of antibiotics in the community to avoid misuse and the contribution to the development of bacterial resistance.

Therefore, the results of the present study highlight the need for promotion and educational policies for the population, with the consequent community benefit that this entails.

## Figures and Tables

**Table 1:** Description of participants.

Gender	N (%)
Male	175 (34.1%)
Female	339 (65.9%)
**Age (years)**	
≤30	150 (29.2%)
30–40	83 (16.1%)
≥40	281 (54.7%)
**Educational level**	
No studies	8 (1.6%)
Primary school	58 (11.3%)
Secondary school	41 (7.9%)
High school	62 (12.1%)
Intermediate vocational training	33 (6.4%)
Higher vocational training	111 (21.6%)
University degree	150 (29.2%)
Postgraduate university degree	47 (9.1%)
Doctorate (PhD)	4 (0.8%)
**Categorization by level of education**	
Low level	107 (28.8%
Medium level	206 (40.1%)
High level	201(39.1%)
**Have you ever had a root canal?**	
Yes	314 (61.1%)
No	200 (38.9%)

**Table 2:** Multivariate regression: final model.

Overall Final Score	Sex		Age			Educational Level		
M ± SD (IC_95_)	Male	Female	≤30	30–40	≥40	Low	Medium	High
6.73 ± 2.34 (6.52–6.93)	6.02 ± 2.39 (5.66–6.38)	7.09 ± 2.23 (6.85–7.33)	6.89 ± 2.41 (6.50–7.28)	6.54 ± 1.81 (6.14–6.93)	6.70 ± 2.44 (6.41–6.98)	6.34 ± 2.29 (5.90–6.78)	6.36 ± 2.30 (6.04–6.68)	7.31 ± 2.29 (6.99–7.63)
*p*-value	0.001		0.521			0.001		
Independent variables	Odds ratio		95% confidence interval			*p*-value		
			Lower		Upper			
Educational level (Ref. Low)	1							
Medium	0.952		0.575		1.557			
High	2.243		1.366		3.684	0.001		
Sex (Ref. Male)	1							
Female	1.988		1.366		2.959	0.001		
Constant	0.300					0.001		

## Data Availability

Data are contained within the article and Supplementary Materials.

## Supplementary Materials

The following supporting information can be downloaded at: https://www.mdpi.com/article/10.3390/jcm13072099/s1, Supplementary S1: Survey on antibiotic consumption in endodontics.
