# Supplementary material for: Patients’ Opinions on Antibiotics in the Treatment of Dental Infections: A Cross-Sectional Survey
Source: J Clin Med. 2024 Apr 3;13(7):2099. doi: 10.3390/jcm13072099 (PMC11012582; doi:10.3390/jcm13072099)
Supplement: Supplementary file 1 [file jcm-13-02099-s001.zip › jcm-2878387-supplementary.pdf]

SURVEY ON ANTIBIOTIC CONSUMPTION IN ENDODONTICS

1. **GENDER:** Man ☐ Woman ☐

2. **AGE (years):** ≤30 ☐ 30-40 ☐ ≥40 ☐

3. **EDUCATIONAL LEVEL:**

☐ - No studies
☐ - Primary school
☐ - High school
☐ - Baccalaureate
☐ - Professional training
☐ - Higher level training cycle
☐ - University degree
☐ - Postgraduate university degree
☐ - Doctorate (PhD)

4. **Have you ever had an endodontic treatment?**

☐ - Yes
☐ - No

5. **Before endodontics, do you think it is necessary to take antibiotics?**

☐ - Yes
☐ - No

6. **After endodontics, do you think it is necessary to take antibiotics?**

☐ - Yes
☐ - No

7. **If the practitioner does not prescribe antibiotics, I would ask why he/she does not prescribe them?**

☐ - Yes
☐ - No

8. **If the practitioner does not prescribe antibiotics, would you seek out another doctor to ask why your doctor does not do so?**

☐ - Yes
☐ - No

9. **If a dentist tells you that you have a dental infection, do you expect him to prescribe antibiotics?**

☐ - Yes

☐ - No

10. **If you suffer from dental pain, do you expect the professional to prescribe antibiotics?**

☐ - Yes

☐ - No

11. **Have you ever self-medicated with antibiotics for dental pain?**

☐ - Yes

☐ - No

12. **What benefits do you think antibiotics have? You can choose more than one option.**

☐ - Decreases pain

☐ - Reduces inflammation

☐ - Decreases infection

☐ - Improves oral health

☐ - Does not provide benefits

☐ - I do not know

13. **What adverse effects do you think the use of antibiotics can cause? You can choose more than one option**

☐ - Nausea/vomiting

☐ - Diarrhea

☐ - Fever

☐ - Fugal infection

☐ - Allergic reaction

☐ - None of the above

☐ - I do not know

14. **When you take antibiotics, for how long do you take them?**

- ☐ - 1 day
- ☐ - 2 days
- ☐ - 3 days
- ☐ - 1 week
- ☐ - 2 weeks
- ☐ - Other

15. **Do you know about "antibiotic resistance" (a process caused by the abusive use of antibiotics)?**

- ☐ - Yes
- ☐ - No

Supplementary S1. Survey on antibiotic consumption in endodontics.
